# G3BP1 modulates SPOP to promote prostate tumorigenesis

**DOI:** 10.1080/23723556.2022.2030171

**Published:** 2022-02-15

**Authors:** Chandrani Mukhopadhyay, Pengbo Zhou

**Affiliations:** Department of Pathology and Laboratory Medicine, Weill Medical College of Cornell University, New York, NY, USA

**Keywords:** G3BP1, SPOP, prostate cancer, CUL3, ubiquitin ligase, androgen receptor

## Abstract

Speckle-type POZ protein (SPOP), a Cullin 3-based ubiquitin ligase (CUL3^SPOP^), acts as a prostate-specific tumor suppressor. Loss-of-function mutations in SPOP occur in 10% of primary prostate cancer with a high Gleason grade and poor prognosis. However, it is unclear how the ubiquitin ligase activity of SPOP is controlled and how dysregulation of SPOP contributes to malignant transformation. Here, we identified GTPase Activating Protein (SH3 Domain) Binding Protein 1 (G3BP1) as an interactor and upstream regulator of CUL3^SPOP^, and it functions as an inhibitor of CUL3^SPOP^ ubiquitin ligase, suggesting a distinctive mode of CUL3^SPOP^ inactivation that aggravates prostate cancer.

Dysregulation of Androgen Receptor (AR) signaling is the major pathway associated with prostate cancer (PCa) progression, and agents targeting AR represent the mainstay of pharmacologic therapy for PCa. Speckle-type POZ protein (SPOP) is a tumor suppressor of PCa and functions as a substrate receptor of the Cullin 3 (CUL3)-based ubiquitin ligase.^1–4^ CUL3^SPOP^ directs ubiquitin-proteasomal degradation of key regulators, e.g., DEK Proto-Oncogene (DEK), Bromodomain Containing 4 (BRD4), Programmed Cell Death Protein 1 (PD1) including AR, and AR co-activators Nuclear Receptor Coactivator 3 (SRC-3) and Tripartite Motif Containing 24 (TRIM24)^1,3,5,6^of the AR signaling pathway. However, the exact mechanisms of SPOP function and regulation in PCa progression remain unclear. Recently, in Nature communications, we have demonstrated a new oncogenic role of stress responsive protein GTPase Activating Protein (SH3 Domain) Binding Protein 1 (G3BP1) that negatively regulates tumor suppressive SPOP ubiquitin ligase, leading to upregulation of AR signaling and prostate tumorigenesis^7^ (Figure 1). The study reveals a new way of SPOP inactivation and provides an opportunity for precision intervention against G3BP1^high^ prostate cancer.

To gain a comprehensive understanding of the SPOP signaling network, we conducted proteomic screen (tandem affinity purification and Mass Spectrometry) for SPOP interactors and identified that G3BP1 binds to and interacts with the SPOP-CUL3 complex (CUL3^SPOP^). To understand the biochemical consequences of G3BP1 and SPOP interaction, we conducted ubiquitination assay where we observed that ectopic expression of G3BP1 reduced CUL3^SPOP^-dependent ubiquitination of AR, a bona fide SPOP substrate. To further understand the molecular mechanism of G3BP1-mediated SPOP suppression, we performed *in vitro* ubiquitination assay with affinity-purified recombinant E1, UBCH5 (as E2), CUL3/RBX1, SPOP, and DEK (substrate) and observed that G3BP1 inhibits CUL3^SPOP^-dependent ubiquitination of DEK in a dose-dependent manner. Moreover, using *in vitro* binding assay with affinity-purified proteins, we further observed that increasing doses of G3BP1 directly competed with DEK for binding to SPOP, suggesting that G3BP1 functions by excluding SPOP substrates resulting in reduced ubiquitination. Thus, it is conceivable that binding of G3BP1 to CUL3^SPOP^ precludes substrate binding and subsequent transfer of ubiquitin to substrates, resulting in increased accumulation of SPOP substrates (such as AR TRIM24 and SRC3) that aggravate PCa progression (Figure 1).

To evaluate the expression and contributions of G3BP1 in PCa patient tissue, we analyzed TCGA RNA sequencing (RNA-seq) data from 498 patient samples and observed that G3BP1 expression is high in higher prostate cancer Grade Group.^8^ Next, we evaluated the prognostic impact of G3BP1 by analyzing the tissue microarray (TMA) of 153 independent PCa cases collected at the Weill Cornell Pathology tumor bank. We observed that G3BP1 expression gradually increased in tissue samples of benign to primary PCa tumors and was most abundant in Castration-resistance prostate cancer (CRPC), suggesting that G3BP1 overexpression is associated with more aggressive disease across the clinical spectrum of prostate cancer. Interestingly, when we evaluated the probability of metastasis-free survival of primary 1,626 PCa patients stratified based on low or high G3BP1 group using genome-wide microarray gene expression data from a clinically available prognostic assay (Decipher; GenomeDx Biosciences, Vancouver, BC, Canada),^9^ we found that patients with ‘‘high’’ G3BP1 expression showed a lesser chance of metastasis-free survival. These findings demonstrated that increased accumulation of G3BP1 corelates with tumor aggressiveness and poor metastasis-free survival.

To provide further insight into G3BP1-mediated deregulation of cellular signaling pathways, we performed RNA-seq of 22RV1 cells after G3BP1 knockout or transient SPOP knockdown and compared them with control cells to identify changes in transcriptional programs. AR signaling output score was determined using a previously defined AR target gene set,^10^ which was found to be decreased in G3BP1-knockout cells and restored upon SPOP knockdown. This was further validated by RT-qPCR using AR target genes. Altogether, these findings suggest that G3BP1 is an upstream regulator of SPOP, and G3BP1-SPOP ubiquitin signaling axis regulates AR threshold levels and transcriptional program. To validate that G3BP1-SPOP axis mediates AR-driven signaling, G3BP1 was overexpressed in wild type primary mPECs (murine prostate epithelial cells) and AR-KO (AR knock out) mPECs. Here, we observed that G3BP1 overexpression (G3BP1^OE^) significantly (p < .001) enhanced organoid formation in WT mPECs, but not AR-KO and AR-KO G3BP1^OE^ mPECs, which establishes a role for AR in G3BP1-SPOP ubiquitin signaling. To understand the clinical implication, we analyzed three PCa data sets and consistently observed that G3BP1 expression directly correlated with AR signaling both at RNA and protein levels. Moreover, we observed that G3BP1 is a bona fide target of AR that upregulates G3BP1 transcription and sets up a feed-forward loop of AR-mediated adverse signaling that further exacerbates PCa progression. These results provide compelling evidence that AR plays a significant role in G3BP1-SPOP axis-mediated downstream signaling in prostate epithelial cells.

Inspired by the findings that the AR signaling pathway is one of the most deregulated pathways by the G3BP1-SPOP ubiquitin signaling axis, we set to investigate its potential role in prostate cancer pathogenesis. We observed that the G3BP1-SPOP axis regulates invasion potential of 22RV1 human prostate cancer cells. As silencing of G3BP1 was observed to reduce invasion, we sought to investigate the proliferative and tumorigenic potential of G3BP1. Stable G3BP1 knockdown in WT (wild type) mPECs significantly reduced organoid formation. Furthermore, G3BP1 knockout significantly attenuated 22RV1 xenograft tumor formation and growth. Therefore, as demonstrated herein, G3BP1^high^ constitutes a distinct subclass of prostate cancer. G3BP1 may serve as a new prognostic biomarker in prostate cancer and provide an opportunity for precision therapy of such G3BP1^high^ patients (Figure 1).

**Figure 1.**
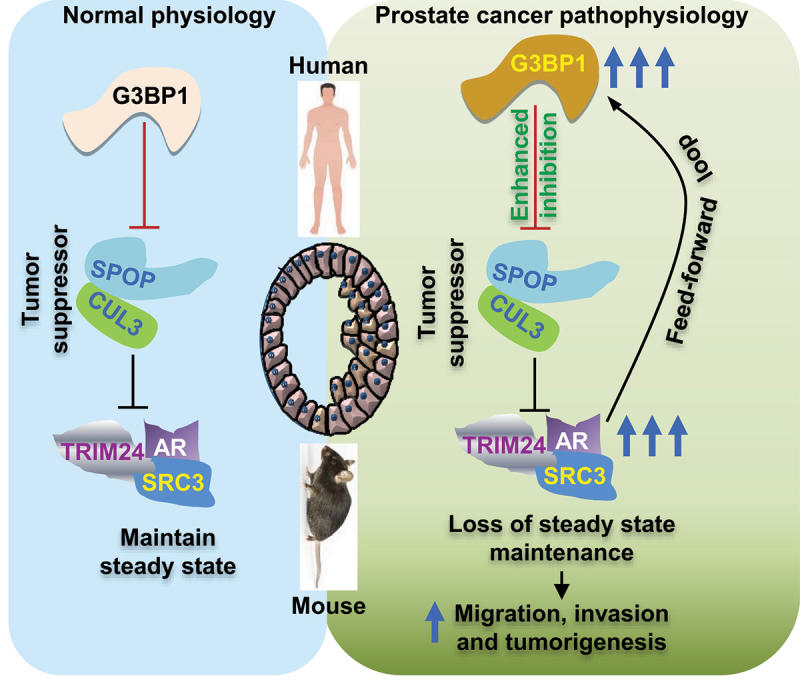
Mechanistic involvement of the G3BP1-SPOP-AR ubiquitin signaling axis in prostate tumorigenesis. Left, in normal physiology, GTPase-Activating Protein (SH3 Domain) Binding Protein 1 (G3BP1) regulates Cullin 3-based ubiquitin ligase (Cul3^spop^) and maintains a steady state level of its substrates in prostate epithelial cells. Right, in prostate cancer pathophysiology, abnormally high expression of G3BP1 inhibits CUL3^SPOP^ function to upregulate (Androgen receptor) AR signaling. Enhanced AR signaling， in turn， upregulates G3BP1 through feed-forward amplification. Upregulated AR signaling enhances the oncogenic phenotype.

**Alt Text** : GTPase-Activating Protein (SH3 Domain) Binding Protein 1 (G3BP1) regulates Cullin 3-based ubiquitin ligase (CUL3^SPOP^) to maintain androgen receptor signaling in normal physiology; however, in prostate cancer pathophysiology, abnormally high expression of G3BP1 inhibits CUL3^SPOP^ function to upregulate AR signaling. Enhanced AR signaling further upregulates G3BP1 and enhances oncogenic phenotype.
